# NAMPT Inhibition Induces Neuroblastoma Cell Death and Blocks Tumor Growth

**DOI:** 10.3389/fonc.2022.883318

**Published:** 2022-06-23

**Authors:** Frederic A. Vallejo, Anthony Sanchez, Branko Cuglievan, Winston M. Walters, Guillermo De Angulo, Steven Vanni, Regina M. Graham

**Affiliations:** ^1^ Department of Neurosurgery, University of Miami Miller School of Medicine, Miami, FL, United States; ^2^ Department of Radiology and Imaging Sciences, University of Utah Hospital, Salt Lake City, UT, United States; ^3^ Department of Pediatrics Patient Care, Division of Pediatrics, The University of Texas MD Anderson Cancer Center, Houston, TX, United States; ^4^ Department of Hematology/Oncology and Immunology, Nicklaus Children’s Hospital, Miami, FL, United States; ^5^ Department of Neurosurgery, HCA Florida University Hospital, Davie, FL, United States; ^6^ Dr. Kiran C. Patel College of Allopathic Medicine, Davie, FL, United States; ^7^ Sylvester Comprehensive Cancer Center, University of Miami Health System, Miami, FL, United States

**Keywords:** NAMPT (Nicotinamide Phosphoribosyltransferase), precision medicine, neuroblastoma, NAD, metabolism, Glycolysis, N-MYC

## Abstract

High-risk neuroblastoma (NB) portends very poor prognoses in children. Targeting tumor metabolism has emerged as a novel therapeutic strategy. High levels of nicotinamide-adenine-dinucleotide (NAD+) are required for rapid cell proliferation. Nicotinamide phosphoribosyl transferase (NAMPT) is the rate-limiting enzyme for NAD+ salvage and is overexpressed in several cancers. Here, we determine the potential of NAMPT as a therapeutic target for NB treatment. NAMPT inhibition cytotoxicity was determined by trypan blue exclusion and LDH assays. Neuroblastoma stem cell self-renewal was evaluated by neurosphere assay. Protein expression was evaluated *via* Western blot. The effect of targeting NAMPT *in vivo* was determined using an NB1691-xenografted mouse model. Robust NAMPT expression was demonstrated in multiple N-MYC amplified, high-risk neuroblastoma cell lines. NAMPT inhibition with STF-118804 (STF) decreased ATP, induced apoptosis, and reduced NB stem cell neurosphere formation. STF treatment down-regulated N-MYC levels and abrogated AKT activation. AKT and glycolytic pathway inhibitors in combination with NAMPT inhibition induced robust, greater-than-additive neuroblastoma cell death. Lastly, STF treatment blocked neuroblastoma tumor growth in mouse xenograft models. NAMPT is a valid therapeutic target as inhibition promoted neuroblastoma cell death *in vitro* and prevented tumor growth *in vivo*. Further investigation is warranted to establish this therapy’s role as an adjunctive modality.

## Introduction

Neuroblastoma (NB) is both the most common extracranial pediatric tumor and the most common occurring cancer in infants under the age of one (1). About 700 cases of NB are diagnosed each year in the United States with median age of diagnosis at 17 months (2). This malignancy is derived from cells in the neural crest lineage in the developing peripheral nervous system, and tumors typically present in the adrenal medulla or paraspinal ganglia (3). The majority of NBs arise sporadically, though 1-2% of NBs have been etiologically associated with ALK, PHOX2B, and KIF1B, mutations (4–6). The clinical manifestations of NBs are very wide ranging from spontaneous tumor regression to extremely deadly, aggressive, metastatic disease (7). The prognosis for high-risk patients is extremely poor, including children with MYCN-amplified tumors, unfavorable tumor biology and/or the presence of distant metastasis (8). About half of NB cases diagnosed are classified as high-risk with long-term survival rates less than 40% (9). Despite an aggressive multi-model treatment regimen including surgery, high-dose chemotherapy, radiotherapy and stem cell transplant followed by retinoid maintenance therapy, children in this cohort often relapse and succumb to the disease and novel therapeutic approaches are desperately required.

**Figure 1:**
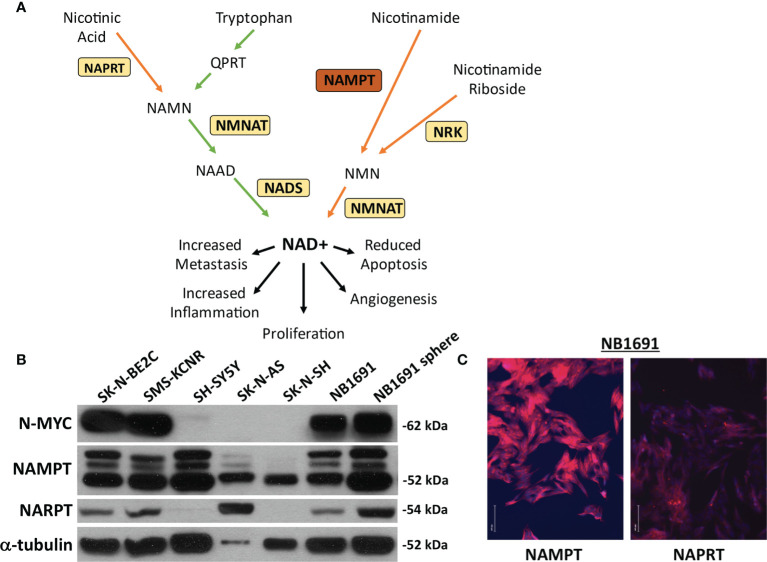
NAMPT and NAPRT expression in NB cell lines. **(A)** Pathway of NAD+ generation. *De novo* pathway denoted with green arrows depicting tryptophan breakdown. Three salvage pathways denoted with orange arrows. Nicotinic acid used as a substrate for Nicotinate phosphoribosyltransferase (NAPRT) and subsequent combination with products downstream of tryptophan prior to final conversion to NAD+ *via* NAD synthase (NADS). Nicotinamide as a substrate utilized by Nicotinamide phosphoribosyltransferase (NAMPT). Nicotinamide riboside is used as a substrate by nicotinamide riboside kinase (NRK). Each pathway requires Nicotinamide-nucleotide adenylyltransferase (NMNAT) prior to conversion to (NAD+). **(B)** Expression of NAMPT, NAPRT and N-MYC were evaluated by western blot assay. **(C)** Immunofluorescence of NAMPT and NAPRT (both in red) in NB1691 cells. Nuclei are stained with DAPI (blue).

It is well established in the literature that many neoplastic cells exhibit Warburg-like increased glycolytic dependency even in the presence of oxygen. Therefore, targeting tumor metabolism and its molecular drivers has recently emerged as a promising therapeutic strategy, potentially causing fewer side effects and enhanced efficacy (10, 11). Because of their high metabolic demands, cancer cells demonstrate increased turnover of nicotinamide adenine dinucleotide (NAD+), a cofactor required for multiple metabolic and cell stress pathways (12, 13). The cell can produce NAD+ either *via* the *de novo* synthesis pathway, utilizing tryptophan as a precursor, or by several salvage pathways using nicotinamide riboside, nicotinic acid, or nicotinamide as substrates. Nicotinamide phosphoribosyl transferase (NAMPT) has been shown to be increased in multiple cancers.This enzyme regenerates NAD+ from nicotinamide and is the rate-limiting enzyme in the salvage pathway of NAD+ generation (**Figure 1A**). NAMPT upregulation has been shown to correlate with worse prognosis as well as conferring “proliferative, anti-apoptotic, pro-inflammatory, pro-angiogenic and metastatic properties” acting as an enzyme, cytokine, and growth factor (14–21). Here we sought to determine the potential of targeting NAMPT, alone and in combination with other metabolic modalities, as a therapeutic strategy for NB.

## Materials and Methods

### Cell Culture and Reagents

The neuroblastoma cell lines SK-N-Be2C, SMS-KCNR, SK-N-SH, SH-SY5Y and SK-N-AS were obtained from American Type Culture Collection (ATCC, Manassas, VA, USA). NB1691 was generously provided by Dr. Andrew Davidoff (St Jude’s Children’s Research Hospital, Memphis, TN, USA). Cell lines were cultured in RPMI-1640 supplemented with 10% heat-inactivated fetal bovine serum and 1% penicillin–streptomycin (penn/strep). NB1691 neurospheres (NB1691-sp) were generated by propagating the cells in 3:1 ratio of Dulbecco’s Modified Eagle’s medium (DMEM): F12 (Gibco) media supplemented with 1% penn/strep, 20 ng/ml human epidermal growth factor and 40 ng/ml human fibroblast growth factor, and 2% Gem21 NeuroPlex Serum-Free Supplement (Gemini Bioscience), a formulation consistent with the growth of neuronally derived cancer stem-like cells (22, 23). For propagation, neurospheres were dissociated using Accutase (Sigma-Aldrich) and reseeded into flasks at 1:5 dilution. Cells were maintained at 37 °C in a humidified 5% CO2 incubator and routinely tested for mycoplasma using LookOut mycoplasma PCR detection kit (SigmaAldrich, St. Louis, MO) according to the manufacturer’s instructions. New cell lines received are routinely expanded with several cryovials frozen down for future use. The NB cell lines utilized are not listed on the most recent NCBI database for misidentification and contamination of human cell lines.

### Immunocytochemistry

Cells were cultured on 4-well plates (Nunc), fixed in 4% paraformaldehyde, washed, blocked and permeabilized with a 5% bovine serum albumin (BSA) with 0.6% Triton-× 100 and then treated with the primary antibodies NAMPT and NAPRT (GeneTex). For analysis of stem cell markers in NB1691-sp cells, cells were plated on laminin/poly-L-Lysine (Sigma-Aldrich) coated plates and treated with Bmi1 or Musashi primary antibodies (Cell Signaling Technology). Cells were then treated with Alexa Fluor 594 -conjugated secondary followed by Prolong Gold Antifade Reagent with DAPI (Thermo Fisher Scientific). Samples were examined under an EVOS FLoid Cell Imaging Station fluorescent microscope (Thermo Fisher Scientific).

### Cytotoxicity

Cytotoxicity was determined using Trypan blue (Sigma-Aldrich) exclusion assay and lactate Dehydrogenase (LDH) assay (Abcam). Stock solutions for the NAMPT inhibitors STF-118804 (Tocris), CHS 828 (Tocris) and FK 866 (Sigma-Aldrich) were made in DMSO, aliquoted and stored in the freezer until use. Apoptosis was assayed *in vitro* using CellEvent™ Caspase-3/7 Green Detection Reagent (Thermofisher Scientific) as per manufacturer’s instructions. Experiments were repeated at least 3 times.

### Neurosphere Formation Assay

The effect of STF on clonogenic growth potential was determined using neurosphere-forming assay, a functional assay that is used to assess the self-renewal capabilities of neural stem cells. Neurospheres were dissociated to single cells and seeded at approximately 25 cells per well in a 96-well plate and treated with varying concentrations of STF on day 0. The number of neurospheres were manually counted under microscopy on day 10. All experiments were done in triplicate.

### Western Blot Assays

Western blot assays were performed as previously described (24). Briefly, proteins (20 μg/sample) were separated using SDS–polyacrylamide gel electrophoresis and electroblotted to nitrocellulose membranes (Bio-Rad). Membranes were probed overnight with the following antibodies: phosphor-AKT (Ser 473), phosphor-AKT (Thr 308), AKT, N-MYC, phosphor-ACC (Ser 79), ACC, phosphor-GSK3b (Ser 9) GAPDH (obtained from Cell Signaling Technology), NAMPT, NAPRT (obtained from GeneTex) or α-tubulin (obtained from Abcam). Membranes were then washed, reacted with horseradish peroxidase–conjugated secondary antibodies and visualized using enhanced chemiluminescence (Thermo Scientific). Relative levels of NAMPT and NAPRT as well as quantitation of cleaved caspase 3 (active) and parp cleavage (indicative of caspase activation) were determined using Image J (imagej.nih.gov).

### ATP Assay

ATP colorimetric assay kit (BioVision) was used to analyze ATP production according to the manufacturer’s protocols. Briefly, cells were lysed in ATP lysis buffer followed by deproteinized using deproteinization kit. Samples were assayed in duplicate and absorbance at 570 nm was measured by using a BioTek Synergy HT Plate reader. ATP levels were determined against an ATP standard curve as per manufacturer’s instructions.

### NB Xenograft

All animal procedures were reviewed and approved by the University of Miami Institutional Animal Care and Use Committee (IACUC, protocol, number 16-083). STF has previously been shown to decrease tumor volume by over 50% compared to vehicle control in *in vivo* tumor models (25). Estimating a conservative effect of 25%, a power of 80% and an alpha level (p value) of 0.05, total number of mice per group would be 8 assuming a standard deviation of 20%. Xenografts were generated as previously described (26). Briefly, NB1691 cells were prepared in using a 1:1 ratio of RPMI and Matrigel (Corning) and 5 x 10^6^ cells injected into the right flank of female nude mice (Charles River Laboratory). Tumor volumes were determined by measuring tumor length and width with calipers and the tumor volume determined using the formula (LxW^2^)/2. When tumors reached an average volume of approximately 180 mm^3^, mice were randomized based on tumor volume into four treatment groups: Vehicle (182 mm^3^, 500mg/kg 2-DG (182 mm^3^), 25mg/kg STF (181 mm^3^), and combined 2-DG and STF (176 mm^3^). 2-DG was prepared in saline and was given intraperitoneal while STF was not soluble in saline and was prepared in DMSO and applied topically to the mouse. The vehicle cohort received both saline and DMSO. Animals were treated daily and tumor volumes and weights were recorded 3 times/week. At end of experiment (when some tumor volumes reached close to 2000 mm^3^) mice were sacrificed and tumors harvested and weighed. Two animals died before completion of study; one had to be sacrificed due to tumor ulcerating (2-DG group) and the other died during injection (STF+2-DG group), leaving 10 mice in the vehicle control and 8 mice in each treatment group. As the primary goal of this experiment was to look at the effect of NAMPT inhibition on tumor progression and tumor volume, only data obtained from animals that completed the experiment were included in the data analysis. To minimize external confounders all animals were housed on the same rack in the same room and received enrichment. Although all researchers were aware of the treatment groups, bias was mitigated by 2 or more researchers being present during treatment and measuring tumor volume/weight.

### Statistical Analysis

Cell viability data were analyzed using Student’s t-tests for all pairwise comparisons of the different treatments that were tested. Single factor ANOVA analyses conducted using Microsoft Excel Analysis ToolPak add-in. The results are presented as the mean ± s.e.m. P-values <0.05 were deemed statistically significant. Bliss Independence Scores were calculated to evaluate potential synergy as previously described (27).

## Results

### NB1691 Chosen as Best Representative Model of High-Risk Neuroblastoma

Neuroblastoma cell lines were assessed for basal expression of N-MYC, NAMPT, as well as nicotinate phosphoribosyltransferase (NAPRT). Four cell lines, SK-N-BE2C, SMS-KCNR, NB1691, and NB1691-sp were found to have amplified N-MYC expression. All seven cell lines demonstrated robust NAMPT expression and variable NAPRT expression (**Figure 1B**). No obvious correlation was noted between N-MYC amplification and NAMPT expression. NB1691 exhibited a higher expression of NAMPT when compared with NAPRT by western blot and when examined on immunofluorescence (**Figure 1C**). Given NB1691’s N-MYC amplification status, high expression of NAMPT and relatively low expression of NAPRT, we chose to focus on this cell line for further investigation.

### NAMPT Inhibition Results in Dose-Dependent Cell Death

**Figure 2:**
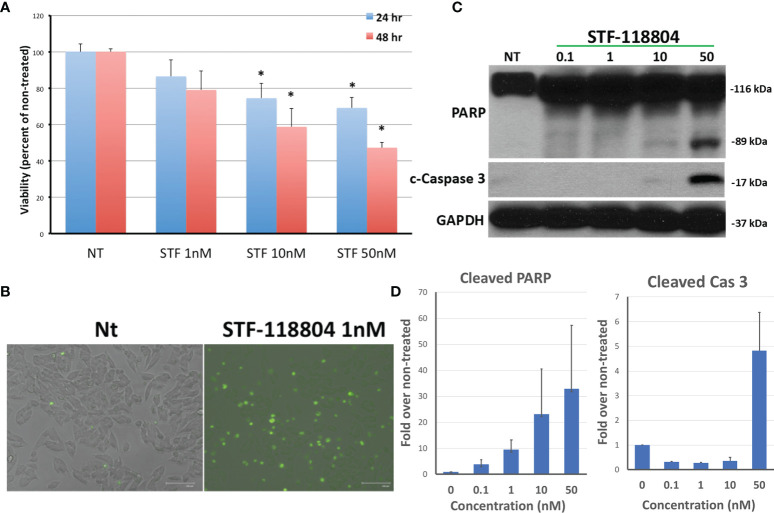
NAMPT inhibition reduces NB1691 cell viability *via* induction of apoptosis. **(A)** NB1691 cells were exposed to the NAMPT inhibitor STF-118804 for 24 and 48 hours at concentrations shown and viability determined using trypan blue assay. **(B)** NB1691 cells were exposed to ST-118804 and caspase 3/7 activity visualized with immunofluorescence. **(C)** Cleaved (active) caspase 3 and cleaved PARP were evaluated by western blot analysis. Alpha-tubulin is used as a loading control. **(D)** Quantification of cleaved PARP and cleaved caspase 3 as compared to non-treated (nM). Experiments repeated in triplicate. *p < 0.05 compared to non-treated controls.

NAMPT inhibitor STF-118804 (STF) was found to induce a dose-dependent reduction in cell viability in line NB1691 after exposure to drug for both 24 and 48 hours. Treating with 10nM STF and above produced a statistically significant reduction in cell viability when compared to non-treated controls (**Figure 2A**). We then sought to understand the mechanism by which NAMPT inhibition was inducing loss of cell viability. Treatment with NAMPT inhibitor STF was found to induce cell death *via* apoptosis as seen on immunofluorescence as well as western blot. Exposure to 1nM of STF induces a robust caspase 3 and caspase 7 activation (**Figure 2B**). Concentrations of STF as low as 0.1nM induced PARP cleavage as evident on Western Blot, indicating that DNA repair was dramatically inhibited at extremely low concentrations. (**Figure 2C**). Quantification of both cleaved PARP and caspase 3 are shown in **Figure 2D**.

### NAMPT Inhibition Reduces Neurosphere Formation

**Figure 3:**
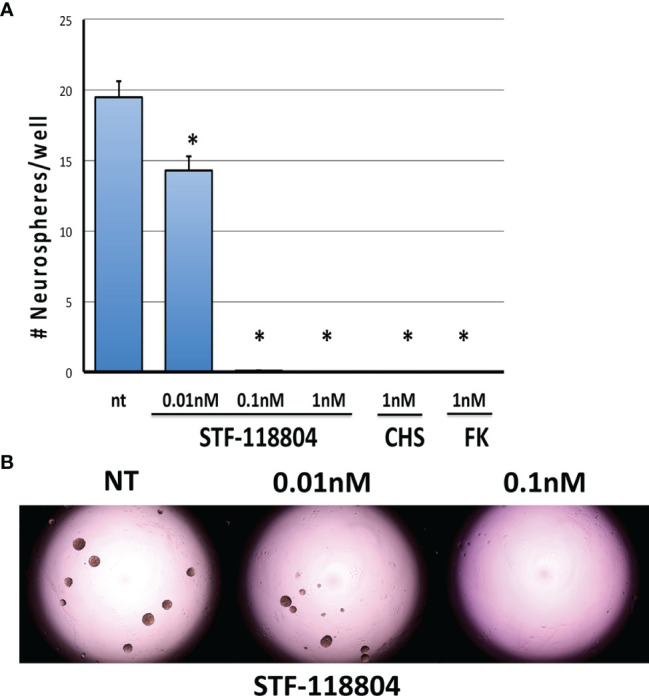
NAMPT inhibition reduces NB1691 cancer stem cell self-renewal. **(A)** NB1691 stem cells were plated at 25 cells/well in 96-well plates and treated with STF-118804 at concentrations shown. Number of neurospheres formed were counted 10 days later. **(B)** Representative light micrographs of NB1691 neurospheres at 10 days. *p < 0.05 compared to non-treated controls.

To select for the NB stem-like cell population in the NB1691 cell line, cells were cultured for several passages in media specifically formulated to promote the growth of neuronally derived tumor stem-like cells. To confirm that our NB1691-sp neurosphere cells expressed stem cell markers the expression of Bmi1 and Musashi was evaluated by immunocytochemistry. Robust expression of both markers was observed. (See **Supplementary Materials**). To evaluate the effect on NAMPT inhibition on the ability of NB stem-like cells to seed new focal adhesions of tumor (analysis of stem cell self-renewal), NB1691-sp neurosphere cells were dissociated and plated as single cells and treated with low concentrations three different NAMPT inhibitors (STF, CHS, and FK), and neurosphere formation assessed 10 days later. STF was found to statistically significantly reduce neurosphere formation at concentration as low as 0.01nM (**Figures 3A, B**). Relatively higher STF concentrations of 0.1nM and 1nM, as well as 1nM concentrations of both CHS and FK, completely eradicated all neurosphere formation after 10 days of drug exposure.

### NAMPT Inhibition Reduces ATP in a Dose-Dependent Manner

**Figure 4:**
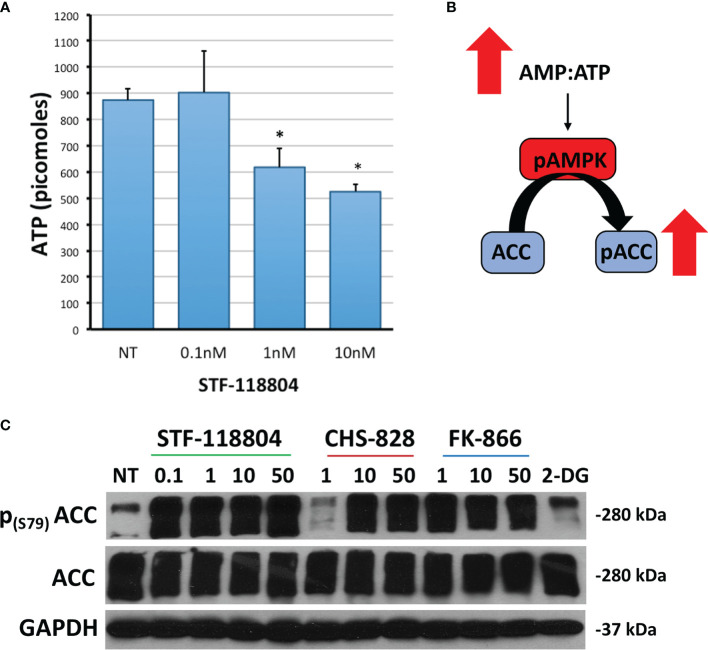
NAMPT inhibition reduces ATP and increases p-ACC levels. **(A)** NB1691 cells were exposed to STF-118804 at concentrations shown and ATP determined using ATP assay. **(B)** Mechanism by which reduction in ATP results in increased phosphorylation of Acetyl Coenzyme-A Carboxylase (p-ACC). **(C)** Levels of p-ACC in response to 24-hour NAMPT inhibition were evaluated by western blot. Alpha-tubulin is used as a loading control. *p < 0.05 compared to non-treated controls.

We then sought to investigate the effect of NAMPT inhibition on ATP production in an effort to better understand how STF would affect broader metabolic pathways. Treating with 1nM STF revealed a statistically significant drop in ATP production (**Figure 4A**). Depletion of ATP also leads to phosphorylation and activation of AMP-activated protein kinase (AMPK). AMPK then phosphorylates Acetyl-CoA carboxylase (ACC) in an attempt to replenish intracellular energy. Thus, phosphorylation of ACC can be used to confirm loss of ATP. Treating with 1nM of STF, CHS, and FK all induced increased expression of p-ACC, and therefore a reduction in ATP, as evident on western blot. STF was noted to significantly induce this effect at an even lower concentration of 0.1nM (**Figures 4B, C**).

### NAMPT Inhibition in Conjunction With PI3K/AKT/Glycolytic Inhibition Maximizes Therapeutic Efficacy *In Vitro*

**Figure 5:**
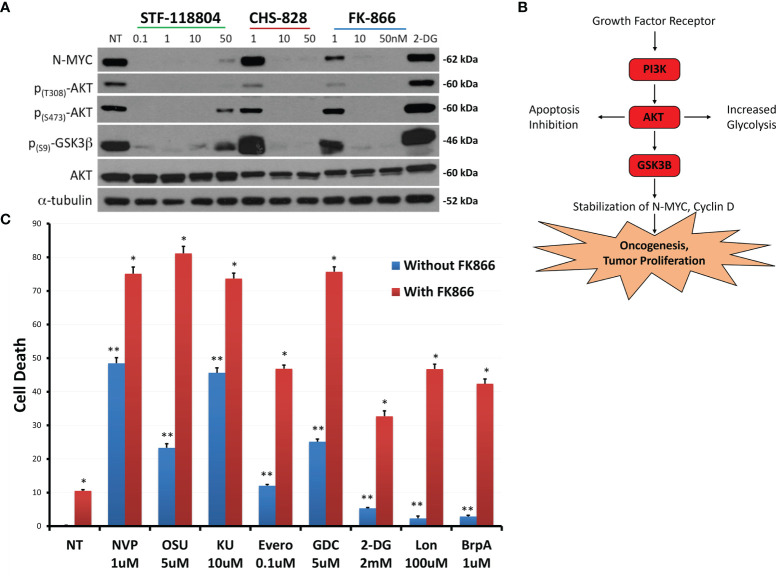
NAMPT inhibition reduces N-MYC levels and AKT pathway activation, and results in greater than additive cytotoxicity when combined with PI3K pathway and glycolytic pathway inhibitors. **(A)** NB1691 cells were treated with NAMPT inhibitors; STF-118804, CHS 828 and FK 866 at concentrations shown, and levels of phospho-AKTs, and N-MYC were evaluated by western blot. **(B)** Growth factors activate PI3K, causing AKT activation, GSK3β sequestration, subsequent stabilization of N-MYC leading to tumor cell proliferation. **(C)** NB1691 cells were pre- treated with10nM FK-866 then exposed to either NVP-BEZ235 (NVP), OSU-03012 (OSU), KU-0063794 (KU), everolimus (Evero), GDC-0941 (GDC), 2-deoxy glucose (2-DG), lonidamine (Lon) or 3-bromopyruvate (3-BrpA) at concentrations shown. Cytotoxicity determined using LDH assay. *p < 0.05 compared to non-treated controls, **p < 0.05 combination drug treatment and FK-866 compared to drug treatment alone.

To further evaluate shifts in molecular proliferation pathways taking place during NAMPT inhibition, we sought to investigate changes in N-MYC expression as well as AKT activation. NB1691 cells were exposed to increasing concentrations of NAMPT inhibitors STF, CHS 828 and FK 866. All three NAMPT inhibitors were found to significantly suppress N-MYC expression as well as halt AKT activation, as evidenced with diminished p-AKT and p-GSK3 levels with drug exposure (**Figures 5A, B**).

Understanding that NAMPT inhibition was affecting downstream mediators of the phosphoinositide 3-kinase (PI3K)/AKT pathway, we sought to see if we could enhance PI3K/AKT/mTOR pathway inhibition to maximize drug-efficacy. Therefore, we investigated if combination treatment with pathway inhibitors could increase cell death when compared with NAMPT inhibition alone. NB1691 cells were treated with either 10nM of NAMPT inhibitor FK-866 alone or in combination with the following inhibitors NVP-BEZ235 (Dactolisib: dual PI3K/mTOR inhibitor), OSU-03012 (AR-12: PDK1/AKT inhibitor), KU-0063794 (mTORC1 and mTORC2 inhibitor) or everolimus (mTORC1 inhibitor). Since NAD+ is necessary for glycolysis, we investigated the effect NAMPT inhibition in combination with the following glycolytic inhibitors: 2-deoxy glucose, lonidamine or 3-bromopyruvate (which all inhibit hexokinase 2) (**Figure 5C**). NAMPT inhibition alone with FK-866 resulted in approximately10% NB cell death as assessed *via* LDH assay following 72 hrs treatment. As solitary treatments, the PI3k/AKT/mTOR inhibitors induced 10-50% depending on the inhibitor, whereas combination treatment resulted in greater-than-additive cell death, inducing between approximately 50%->80% cell death. The glycolytic inhibitors alone induced very little cell death (< 10%), however when combined with FK-866, cell death increased to approximately 30%-50% cell death. This robust increase in cell death with combined therapy may suggest potential synergy between NAMPT and PI3K/AKT/mTOR inhibition as well as with glycolytic inhibition (**Supplementary Table 1**).

### NAMPT Inhibition Halts Tumor Growth *In-Vivo*

**Figure 6:**
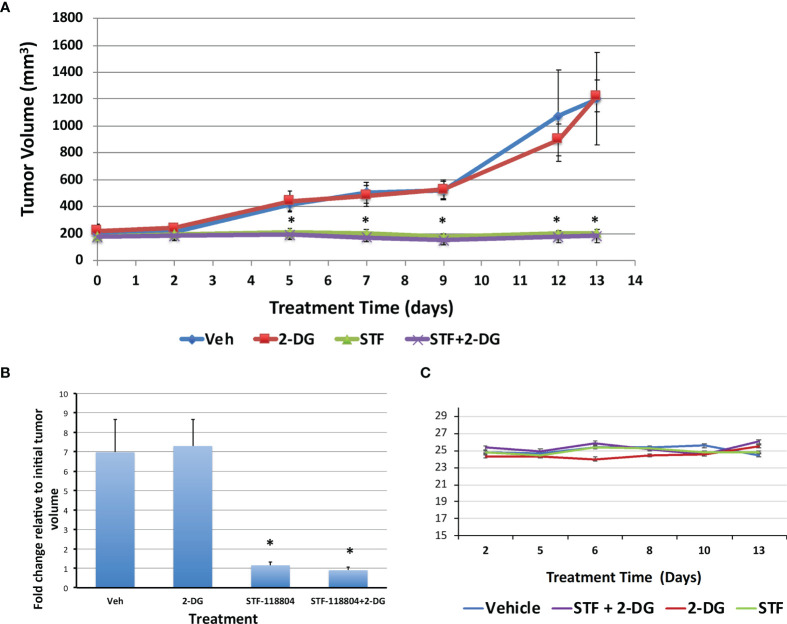
NAMPT inhibition blocks tumor growth in NB1691 xenograft model. NB1691 cell were injected into the flank of nude mice and when tumors reached approximately 200mm^3^ (treatment day 0), mice were treated with either vehicle, 25mg/kg STF-118804 (STF), 500mg/kg 2-DG (2-DG) or 25mg/kg STF-118804 + 500mg/kg 2-DG (STF+2-DG) daily. All mice were euthanized on day 13 of treatment. **(A)** Tumor volume over treatment period. **(B)** Fold change in tumor volume relative to initial tumor volume. **(C)** Average weight of each experimental group over course of treatment. *p<0.05 compared to vehicle-treated controls.

After we established the efficacy of NAMPT inhibition on our *in-vitro* experiments, we wanted to verify the feasibility and validity of NAMPT inhibition in an *in-vivo* xenograft murine model. To determine the effect of NAMPT inhibition on tumor growth, NB1691 cells were injected into the flank of nude mice and allowed to grow until reaching a tumor volume of approximately 200mm^3^. Mice were then split into four treatment arms including control, STF alone, 2-DG alone, or combination STF plus 2-DG. Mice were treated each day for 2 weeks with tumor volume measurements taken 3X/week to monitor progression. Both the control group and the 2-DG alone group experienced significant tumor growth over the course of two weeks, increasing from approximately 200mm^3^ to 1200mm^3^ before being put down with euthanasia. There was no significant difference between the control and 2-DG alone experimental arms. The two groups treated with STF experienced dramatically less tumor progression and growth (**Figures 6A, B**). ANOVA analysis confirmed a significant difference between mean tumor volume growth (p=0.0088) and no significant difference between average mouse weights (p=0.053) over the experimental period (**Figure 6C**). Both the STF and STF+2-DG groups benefitted from stable, progression-free tumors as evidenced by the consistent tumor volume over the course of treatment. Although the combination group did have tumor volumes that trended lower than STF alone, this difference was not statistically significant.

## Discussion

This project sought to investigate the feasibility and efficacy of NAMPT inhibition, alone and in combination with other metabolic modalities, as a therapeutic approach to treating NB. Studies thus far have predominantly investigated NAMPT inhibitors as few NAPRT and Nicotinamide-nucleotide adenylyltransferase (NMNAT) inhibitors have been identified to date. Many NAMPT inhibitors are currently under investigation, and several are currently being tested in clinical trials (19, 28, 29). Common side effects reported in these trials thus far include thrombocytopenia, diarrhea, mucositis, anemia, and leukopenia. We elected to use STF-118804 for our *in-vivo* experimentation as it showed promising results as a NAMPT-specific inhibitor *in-vitro*, has not been explored as thoroughly in the literature, and has been shown to sensitize tumor cells to chemotherapeutic agents (25, 30). STF acts to competitively inhibit NAMPT, inducing apoptosis and cell cycle arrest (31). NAD is necessary for a multitude of intracellular processes, NAMPT inhibition therefore is closely associated with changes in other cellular and metabolic pathways. NAMPT has been shown to increase neoplastic cells’ ability to tolerate reactive oxygen species, and NAMPT inhibition has been shown to increase intracellular ROS and enhance susceptibility to oxidative stress (32). Though NAMPT inhibitors have demonstrated therapeutic efficacy when delivered alone *in vitro*, evidence suggests that these drugs may be more effective when used in combination with other therapies (33).

Varying proteins found in several tumor types in other molecular pathways have recently emerged as potential biomarkers to indicate which candidates may benefit the most from NAMPT inhibition. Therefore, establishing expression levels of enzymes specific to both NAD generation pathways in each unique tumor is of utmost importance when designing future experiments or precision therapies for patients. Tumors which have upregulated the salvage pathway *via* NAMPT overexpression would be the best candidates for therapies such as STF, whereas tumors expressing high levels of *de-novo* pathway enzymes or NAPRT would be poorer candidates for NAMPT inhibition. Both *in-vitro* and clinical data have established an interesting inverse relationship between NAMPT expression and the expression of p73, a tumor suppressor across multiple malignancies. Low levels of p73 expression correlate with poorer response to NAMPT inhibition, whereas upregulation of p73 enhances the efficacy of NAMPT-targeted therapy (34). Recent work has demonstrated that N-MYC amplified gliomas, exerting dramatically increased dependence on glycolysis, may be particularly susceptible to NAMPT inhibition as an anti-metabolic therapy (35, 36). This effect is not limited to glioblastoma, as any tumor with amplification of N-MYC may exhibit this onco-metabolic vulnerability. Additionally, IDH1, R132H, and PPM1D mutant gliomas have been shown to selectively rely on NAD salvage pathways, suggesting these sub-populations of tumors may benefit from NAMPT inhibition (37–39).

Different neoplasms have exhibited unique expression profiles which may indicate how sensitive each unique tumor would be to NAMPT inhibition. A recent study showed that in high-grade pediatric gliomas such as DIPG, a protein phosphatase coined PPM1D is commonly truncated. The absence of this functioning protein acts to drive CpG island hypermethylation throughout the genome, specifically knocking out transcription and expression of NAPRT (38). Thus, PPM1D mutation could act as a biomarker to stratify future candidates who would experience dramatic intra-tumoral NAD depletion with NAMPT inhibition, while also benefitting from minimal peripheral toxicity as the *de-novo* pathway would be preserved in non-malignant cells. Interestingly, PPM1D mutant DIPG have been shown to be sensitive to PARP inhibition as well, emphasizing the importance of combining these novel therapies to shut pathways off at multiple levels to avoid escape (40). IDH1/2 mutant tumors such as glioblastomas, chondrosarcomas, and gastric cancers have also been shown to be more sensitive to NAMPT inhibition. This effect is believed to be due to hypermethylation of the NAPRT promoter by D-2hydroxyglutarate, a byproduct of α-ketoglutarate’s interaction with the aberrant enzyme (41, 42).

Effective inhibition of NMNAT, the enzyme at which both pathways converge, would theoretically ensure global rapid NAD depletion. One concern with this strategy, however, would be therapy-limiting toxicity to peripheral tissues which are also actively metabolizing and dependent on NAD. One study demonstrated slower NB progression when treated with daily 20mg/Kg Vacor, an NMNAT2 inhibitor previously used as a rodenticide, when compared with control mice (43). This effect was elicited only in NB cells which were found to express NMNAT2. In these cells, products of Vacor metabolism were shown to deplete NAD stores as well as inhibit NAD-dependent dehydrogenases such as GAPDH, leading to necrotic cell death. Similarly, another group found that STF-31 may be acting as both a NAMPT inhibitor as well as a GLUT inhibitor at higher concentrations (44). As previously stated, Tateishi et al. demonstrated that N-MYC amplified patient-derived glioblastoma orthotopic xenograft mice exhibited heightened glycolytic dependence and increased susceptibility to NAMPT inhibition (35). This group went on to show that multiple N-MYC amplified neoplasms including lung cancer, medulloblastoma, neuroblastoma, and Burkitt’s lymphoma were susceptible to NAMPT inhibition with GMX1778 at nanomolar concentrations. When N-MYC was knocked down *via* shRNA, however, the cells were not as susceptible to the treatment and NAD+ levels rose intracellularly. N-MYC amplification in aggressive NBs may also stratify future patients to receive adjunctive metabolic and molecular therapies alongside current standard of care (45). Taken together, these findings further underscore the close relationship between therapies targeting NAD depletion such as NAMPT inhibition, and therapies aiming to shut down aerobic glycolysis such as glycolytic inhibition.

Here, we showed that NAMPT inhibition with STF reduces ATP and promotes PARP cleavage with subsequent caspase-cascade activation. This therapy was shown to reduce cell viability significantly at concentrations as low as 10nM and disrupt colony formation at concentrations of 0.01nM in NB1691 cells, suggesting this therapy may be viable to target NB stem-like cells. NAMPT inhibition was shown to downregulate both AKT and N-MYC activity while maximizing therapeutic efficacy when delivered in conjunction with glycolytic and PI3K pathway inhibitors. This metabolic stress induced by NAMPT inhibition was apparent with p-ACC activation as a potential mechanism by which to replete NAD+ stores (46). Lastly, NAMPT inhibition, alone or in conjunction with glycolytic inhibition, was shown to significantly halt tumor growth *in-vivo*.

Given our group’s previous work with 2-DG showing synergistic interactions with other anti-tumoral therapies in addition to the drugs’ well-tolerated safety profile when compared to the other glycolytic inhibitors in previous clinical trials, we elected to use 2-DG alongside NAMPT inhibition in our xenograft models (24, 47–50). Though the combination treatment group STF+2-DG had tumor volumes trending smaller than those treated with STF alone, this difference was not statistically significant. We posit that the observed greater-than additive effect apparent *in vitro* was not demonstrated *in vivo* due to the mice’s continuous availability of food, allowing the mice to maintain itself in a satiated, hyperglycemic state minimizing the glycolytic flux of 2-DG at the tumor site. This may be mitigated in future experiments by administering the drug after a period of fasting, as is clinically routine with positron emission topography (PET) scans to maximize localization of fluorodeoxyglucose (F-DG) to cancerous cells. Alternatively, other inhibitors of glycolysis may prove to be a better therapeutic option. For example, ACT-PFK-158, a 3-(3-pyridinyl)-1-(4-pyridinyl)-2-propen-1-one (3PO) derivative is currently in phase 1 clinical trials for solid tumors. 3PO and it’s derivative inhibit 6‐phosphofructo‐2‐kinase/fructose‐2,6‐biphosphatase 3 (PFKFB3), an important regulator of glycolysis.

It remains to be seen what the role of NAD depletion therapy will be in the future. Although our *in vivo* results are promising, a limitation noted in this study was the use on only one cell line for *in vivo* testing, therefore, additional *in vivo* studies using cell lines with variable NAMPT and NAPRT levels are needed to fully understand the impact of NAMPT targeting in NB. Further studies should be aimed at finding biomarkers to stratify which patients would be the best candidates for NAMPT inhibition. By assessing expression profiles of tumors in real-time, clinicians can better tailor which treatments would work best in combination with NAMPT inhibition. Inhibition of NAMPT could be used alongside traditional chemo and radiotherapy and in conjunction with glycolytic inhibition, PARP inhibition, PI3K pathway modulators, and ROS inducers. Future studies should aim to maximize the synthetic lethality of NAD depletion and minimize peripheral toxicity. This can be achieved by multiple means including but not limited to drug-delivery systems and using lower concentrations with synergistic combination therapies. Just as the ketogenic diet has been proposed to induce glucose starvation to increase glycolytic inhibitor efficacy, so too can patients receiving NAMPT inhibitors limit consumption of NAD precursors like tryptophan and Niacin consumption in an effort to maximize NAMPT inhibitor efficacy (51). Further studies are warranted to optimize combination therapies and evaluate synergistic interactions more fully. NAPRT inhibition may serve as a promising future tool to use if tumors develop resistance to NAMPT inhibition or if NAPRT is upregulated. Additionally, tumors which initially respond to NAMPT inhibition alone that potentially develop resistance as the treatment regimen continues may benefit from targeting the opposite arm of the NAD generation pathway.

## Data Availability Statement

The original contributions presented in the study are included in the article/**Supplementary Material**. Further inquiries can be directed to the corresponding author.

## Ethics Statement

The animal study was reviewed and approved by the University of Miami IACUC Committee.

## Author Contributions

FV and RG conducted *in vitro* and *in vivo* experiments, drafted, and edited manuscript. RG also directed experimental design and supervised research team. AS and BC assisted with *in vitro* experiments as well as drafting and editing manuscript. GD and SV assisted in experimental design as well as drafting and editing manuscript. WW assisted in invitro and *in vivo* experimentation. All authors contributed to the article and approved the submitted version.

## Funding

We would like to thank the Mystic Force Foundation, Cancer Free Kids Foundation, and Florida Department of Health Live Like Bella Pediatric Research Initiative (Grant number: 21L08) for providing salary support for RG and cost of all materials/reagents required for this work.

## Conflict of Interest

The authors declare that the research was conducted in the absence of any commercial or financial relationships that could be construed as a potential conflict of interest.

## Publisher’s Note

All claims expressed in this article are solely those of the authors and do not necessarily represent those of their affiliated organizations, or those of the publisher, the editors and the reviewers. Any product that may be evaluated in this article, or claim that may be made by its manufacturer, is not guaranteed or endorsed by the publisher.

## Abbreviation

NB, Neuroblastoma; NAD+, nicotinamide adenine dinucleotide; NAMPT, Nicotinamide phosphoribosyl transferase; NAPRT, nicotinate phosphoribosyltransferase; NMNAT, Nicotinamide-nucleotide adenylyltransferase; NADS, NAD Synthase; NRK, Nicotinamide riboside kinase; ATP, Adenosine Triphosphate; LDH, Lactate dehydrogenase; GAPDH, Glyceraldehyde 3-phosphate dehydrogenase; p-ACC, Phospho-Acetyl-CoA Carboxylase; DAPI, 4′,6-diamidino-2-phenylindole; STF, STF-118804; CHS, CHS 828; FK, FK-866; NVP, NVP-BEZ235; OSU, OSU-03012; KU, KU-0063794; GDC, GDC-0941; Lon, Lonidamine; BrpA, 3-bromopyruvate; Evero, Everolimus; GSK3b, Glycogen synthase kinase-3β; DIPG, Diffuse intrinsic pontine glioma; IDH, Isocitrate Dehydrogenase; PET, positron emission topography; PFKFB3, phosphofructo‐2‐kinase/fructose‐2,6‐biphosphatase 3; F-DG, fluorodeoxyglucose; 2-DG, 2-deoxy D glucose; 3PO, 3-(3-pyridinyl)-1-(4-pyridinyl)-2-propen-1-one

## Glossary

NB: Neuroblastoma
NAD+: nicotinamide adenine dinucleotide
NAMPT: Nicotinamide phosphoribosyl transferase
NAPRT: nicotinate phosphoribosyltransferase
NMNAT: Nicotinamide-nucleotide adenylyltransferase
NADS: NAD Synthase
NRK: Nicotinamide riboside kinase
ATP: Adenosine Triphosphate
LDH: Lactate dehydrogenase
GAPDH: Glyceraldehyde 3-phosphate dehydrogenase
p-ACC: Phospho-Acetyl-CoA Carboxylase
DAPI: 4″6-diamidino-2-phenylindole
STF: STF-118804
CHS: CHS 828
FK: FK-866
NVP: NVP-BEZ235
OSU: OSU-03012
KU: KU-0063794
GDC: GDC-0941
Lon: Lonidamine
BrpA: 3-bromopyruvate
Evero: Everolimus
GSK3b: Glycogen synthase kinase-3β
DIPG: Diffuse intrinsic pontine glioma
IDH: Isocitrate Dehydrogenase
PET: positron emission topography
PFKFB3: phosphofructo–2–kinase/fructose–2,6–biphosphatase 3
F-DG: fluorodeoxyglucose
2-DG: 2-deoxy D glucose
3PO: 3-(3-pyridinyl)-1-(4-pyridinyl)-2-propen-1-one
