# Supplementary material for: NAMPT Inhibition Induces Neuroblastoma Cell Death and Blocks Tumor Growth
Source: Front Oncol. 2022 Jun 23;12:883318. doi: 10.3389/fonc.2022.883318 (PMC9261286; doi:10.3389/fonc.2022.883318)
Supplement: Supplementary file 1 [file DataSheet_1.pdf]

Supplemental Figure 1.

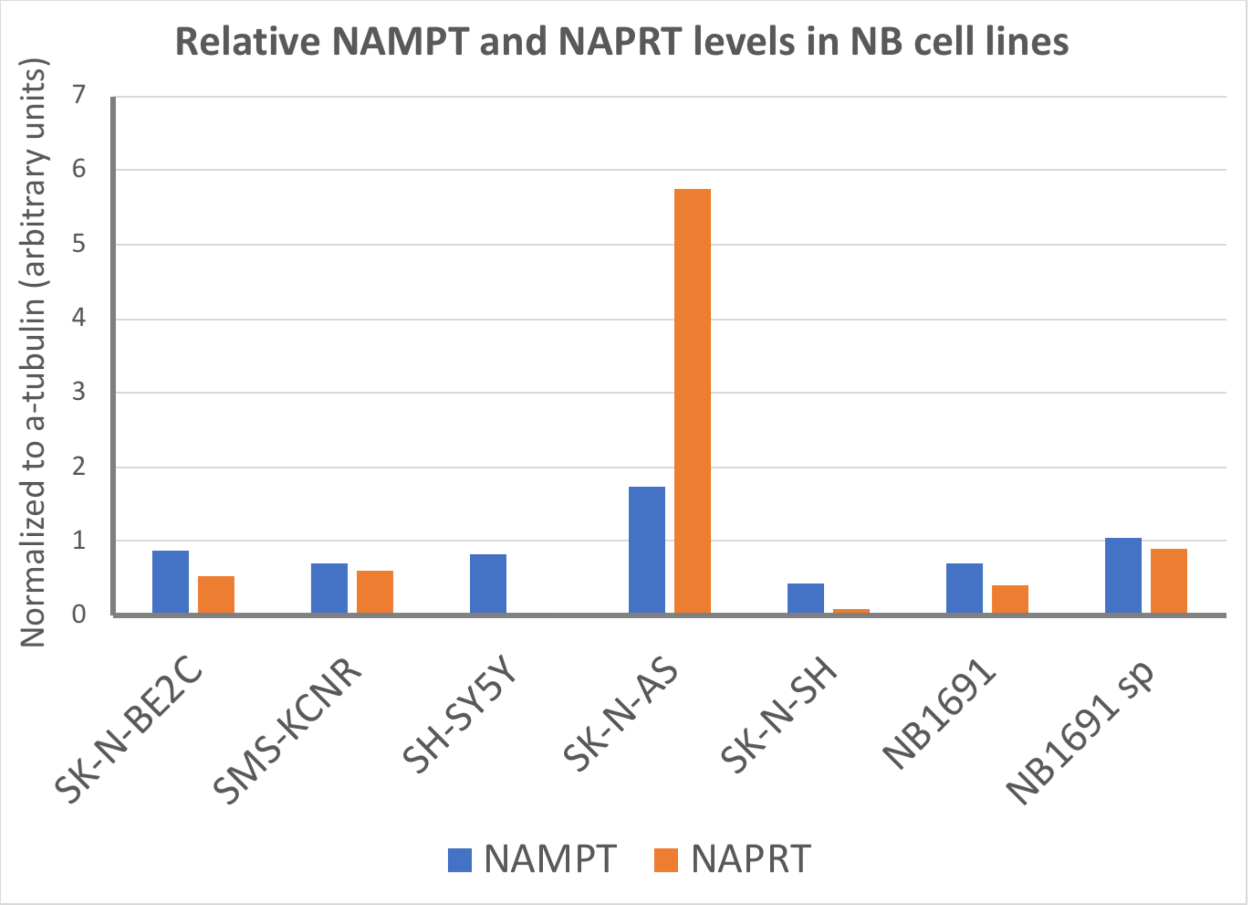

Supplementary Figure 1. The levels of NAMPT and NAPRT proteins depicted in Figure 1B were normalized to □-tubulin in all cell lines examined. NB1691 neurosphere cells depicted as NB1691-sp.

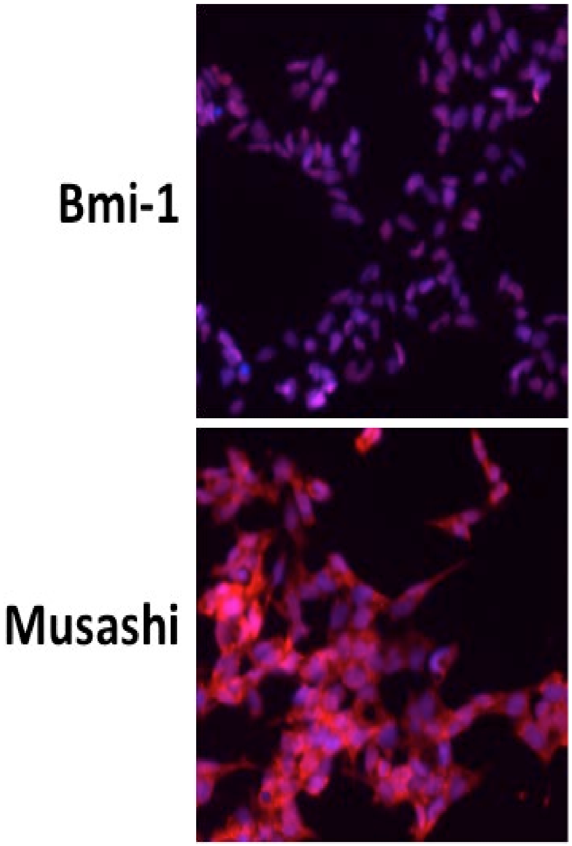

Supplementary Figure 2. Immunofluorescence of NB1691 neurosphere cells demonstrating presence of stem cell markers.

Supplementary Table 1. Bliss Independence Scores for Synergy detection. In reference to Figure 5C.

	Y(a)	Y(b)	Y(a)Y(b)	Y(ab) predicted	Y(ab) actual	Excess over Bliss Score	Synergy Observed? >0?
FK866	10.52607	n/a	n/a	n/a	n/a	n/a	n/a
NVP 1uM	0.105261	0.484776	0.051028	0.539009	0.751369	0.212359	Yes
OSU 5uM	0.105261	0.233278	0.024555	0.313984	0.811757	0.497773	Yes
KU 10uM	0.105261	0.45633	0.048034	0.513557	0.736813	0.223257	Yes
Evero 0.1uM	0.105261	0.120493	0.012683	0.21307	0.468389	0.255319	Yes
GDC 5uM	0.105261	0.251181	0.02644	0.330002	0.757128	0.427126	Yes
2-DG 2mM	0.105261	0.053126	0.005592	0.152795	0.327128	0.174333	Yes
Lon 100uM	0.105261	0.023165	0.002438	0.125988	0.467542	0.341555	Yes
BrpA 1uM	0.105261	0.028709	0.003022	0.130947	0.423639	0.292691	Yes
